# Gene Expression Profiling in Lungs of Chronic Asthmatic Mice Treated with Galectin-3: Downregulation of Inflammatory and Regulatory Genes 

**DOI:** 10.1155/2011/823279

**Published:** 2011-03-20

**Authors:** Esther López, M. Paz Zafra, Beatriz Sastre, Cristina Gámez, Carlos Lahoz, Victoria del Pozo

**Affiliations:** Immunology Department IIS-Fundación Jiménez Díaz, CIBER de Enfermedades Respiratorias (CIBERES), 28040 Madrid, Spain

## Abstract

*Background*. Asthma is a disorder characterized by a predominance of Th2 cells and eosinophilic inflammation. Suppressors of cytokine signaling (SOCS) proteins act as negative regulators of cytokine signaling. In particular, SOCS1 and SOCS3 play an important role in immune response by controlling the balance between Th1 and Th2 cells. In a previous study, we demonstrated that treatment of chronic asthmatic mice with gene therapy using plasmid encoding galectin-3 (Gal-3) led to an improvement in Th2 allergic inflammation. *Methods*. Using a microarray approach, this study endeavored to evaluate the changes produced by therapeutic Gal-3 delivered by gene therapy in a well-characterized mouse model of chronic airway inflammation. Results were confirmed by real-time RT-PCR, Western blot and immunohistochemical analysis. *Results*. We identify a set of genes involved in different pathways whose expression is coordinately decreased/increased in mice treated with Gal-3 gene therapy. We report a correlation between Gal-3 treatment and inhibition of SOCS1 and SOCS3 expression in lungs. *Conclusion*. These results suggest that negative regulation of SOCS1 and 3 following Gal-3 treatment could be a valuable therapeutic approach in allergic disease.

## 1. Introduction

Asthma is a complex chronic disease characterized by airway inflammation, airway hyperresponsiveness (AHR), and reversible airway obstruction. In addition, structural changes causing remodeling occur as a result of an imbalance in the mechanisms behind lung tissue regeneration and repair [1, 2].

Cytokines play an essential role in immune system regulation [3]. In both physiologic and pathologic conditions, cytokine functions are strictly controlled. Cytokine signaling pathways are negatively regulated by the so-called suppressor of cytokine signaling (SOCS) family of proteins. SOCS proteins not only act as simple negative-feedback regulators, but are also involved in fine-tuning the immune response and in the cross-talk of the complicated cytokine signal networks [4]. Since cytokines are constantly present in the microenvironment of immune cells, signal regulation by SOCS-family proteins must be important for the proper progress, remission, and relapse of an immune response. SOCS1, SOCS3, and SOCS5 therefore participate in CD4+ Th-cell differentiation and in Th1/Th2-cell balance [5]. Of these, SOCS3 has been shown to inhibit IL-2 production during T-cell activation, is predominantly expressed in Th2 cells, and inhibits Th1 differentiation [6–8]. Conversely, SOCS5 is predominantly expressed in Th1 cells and inhibits Th2 differentiation [9]. The data with respect to SOCS1 are controversial; indeed, Fujimoto et al. showed that SOCS1 negatively regulates both Th1 and Th2 cell differentiation in response to IL-12 and IL-4, respectively [10]. In contrast, SOCS1 is a strong negative-feedback regulator of IFN*γ*, and thus positively regulates Th17-cell differentiation by suppressing the antagonistic effect of IFN*γ* [4]. 

Gal-3 is an immunoglobulin IgE-binding protein which belongs to a family of proteins that bind *β*-galactosides. It has a unique amino-terminal domain, a highly conserved repetitive sequence rich in proline and glycine, and a globular carboxyl-terminal domain containing the carbohydrate recognition site. Gal-3 is expressed in a variety of tissues and cell types [11]. In addition, it has been implicated in different processes, including inflammation and allergic pathologies [12, 13]. We previously reported that administration of Gal-3 by means of gene therapy inhibits asthmatic reactions by downregulating IL-5 gene expression [14–16]. However, in a complex pathology such as asthma, there are many factors and pathways implicated, which is why we planned to study more genes. 

Genome-wide expression analysis with microarrays has become a mainstay of genomics research [17, 18]. The challenge no longer lies in obtaining gene expression profiles, but rather in interpreting the results to gain insights into biological and molecular disease mechanism or identify gene expression changes characteristic of different diseases, or to study the gene expression profiles, whose are affected by specific treatment. To overcome these analytical challenges, several authors propose evaluating microarray data at the gene-set level-based on prior biological knowledge [19]. 

In this study, we used cDNA microarrays to gain a detailed molecular picture of the programmed responses of the asthmatic mice to Gal-3 gene therapy. The transcriptional program was analyzed in lung samples from asthmatic mice treated with Gal-3 and asthmatic mice that did not receive the treatment. A set of pathways and genes was identified by microarrays and then selected for further analysis by RT-PCR, immunohistochemistry staining, and Western blot to confirm and explore their corresponding protein level. Analysis of the transcriptional program reveals striking evidence of global differences between mice treated with Gal-3 and those that are not. The most prominent feature of the gene signature identified in treated mice was a pattern of reduced inflammatory genes and regulatory genes. SOCS1 and 3 were also among the inflammatory genes found to be more abundant in asthmatic mice as compared to Gal-3-treated mice. Accordingly, one of the new models of therapy for allergic diseases could entail targeting SOCS3 by using a Gal-3 gene therapy approach via inhibition of Th2 response. Such an immunomodulatory approach may have the beneficial effect of balancing the immune system.

## 2. Materials and Methods

### 2.1. Animals and Development of Chronic Asthma by Intranasal Administration of OVA and Gal-3 Gene Treatment

All experimental procedures conformed to international standards of animal welfare and were approved by the Fundación Jiménez Díaz Animal Research Ethics Committee. Male A/J mice (specific pathogen-free: 5 weeks old) were purchased from Harlan Iberica.

Experimental protocols were performed as previously reported [16]. The mice were placed in a small box and anesthetized with inhaled Forane (Abbott, IL). The anesthetized mice inhaled 1 mg/mL OVA (grade V; Sigma, St. Louis, MO) intranasally. OVA was administered 3 days per week for twelve weeks using a previously described method [16]. 

Fourteen days after the first OVA inhalation and once every 15 days thereafter, the mice received intranasal inhalations of 50 *μ*L of plasmid (1 mg/mL) encoding Gal-3 (pEGFP-Gal-3, *n* = 20) or with plasmid without insert (pEGFP, *n* = 15) or with saline (OVA, *n* = 15) as positive controls. An additional negative control group was used in which mice were injected with saline and exposed to saline inhalation (SS, *n* = 10).

After instillation of plasmid into the lungs, Gal-3 was detected by immunohistochemistry as previously reported [16].

### 2.2. RNA Extraction and Microarray Hybridization

Twenty-four hours after the last administration of antigen, the mice were anesthetized and the lungs were removed. Total RNA was isolated from the lungs using TRIzol Reagent (Life Technologies, Paisley, UK) according to the manufacturer's protocol. Purity and integrity of the RNA was assessed using an Agilent 2100 Bioanalyzer (Agilent Technologies, Palo Alto, CA, USA), and degraded samples were discarded. Concentration was determined using a Nanodrop ND-1000 spectrophotometer. A total of 5 *μ*g of purified RNA samples was submitted to the Gene Expression Department of Complutense University (Madrid, Spain) for labeling and hybridization. In total, 19 samples were analyzed: six from the OVA group, 8 from the pEGFP-Gal-3 group, and five from the SS group. Following all appropriate protocols and procedures for quality control, labeling, and fragmentation of eukaryotic total RNA, the biotin-labeled cRNA samples were hybridized to Mouse Genome 430 2.0 Microarrays (Affymetrix, Santa Clara, CA) according to manufacturer protocols.

### 2.3. Microarray Data Analysis

#### 2.3.1. Bioinformatics Analysis

Microarray analysis was performed by the Bioinformatics Department of the Centro de Investigación Príncipe Felipe (Valencia, Spain) using GEPAS version 3.1 (http://www.gepas.org/) [20]. Functional analysis was carried out using the Babelomics suite (http://www.babelomics.org/).

#### 2.3.2. Preprocessing

Output data from the microarray normalization process were preprocessed before micrroarray analysis. Data were standardized using RMA [21]. Multiple probes mapping to the same gene were merged using the average as the summary of the hybridization values.

#### 2.3.3. Differential Gene Expression

Differential gene expression was carried out using the *Limma *package from Bioconductor (http://www.bioconductor.org/) [22]. To account for multiple testing effects, *P*-values were corrected using the false discovery rate [23].

#### 2.3.4. Functional Analysis

FatiScan, a variant of the gene set enrichment algorithm that detects significant up- or downregulated blocks of functionally related genes from a list of genes ordered by differential expression [24], was used to detect activations or deactivations in biological functions or pathways. FatiScan is part of the Babelomics suite. FatiScan can search blocks of genes that are functionally related by different criteria such as gene ontology terms or KEGG pathways.

### 2.4. Real-Time RT-PCR

Quantitative real-time PCR was performed for selected genes (SOCS-1, -3, -5, IL-10, and TGF-*β*1) on a 7500 Real-Time PCR System (Applied Biosystems, Warrington, UK). TaqMan PCR was performed using a 20 *μ*L final reaction volume containing 10 *μ*L of TaqMan Universal PCR Master Mix (Applied Biosystems, Branchburg, NJ, USA), 1 *μ*L of 20X Assays-on-Demand Gene Expression Assay Mix, and 9 *μ*L of cDNA diluted in RNase-free water. Each assay was performed in triplicate. The PCR conditions used in all reactions were 2 min at 50°C and 10 min at 95°C, with 40 two-step cycles (95°C for 15 s and 60°C for 60 s). Assays-on-Demand Gene Expression primers specific for SOCS1, SOCS3, SOCS5, IL-10 and TGF-*β*1 and rRNA (used as an endogen) were obtained from Applied Biosystems (http://www.appliedbiosystems.com/). The genes analyzed in this study were examined for their relative expression by means of the ΔΔC_T_ method [25].

#### 2.4.1. Western Blot Analysis

Lung lysates were prepared in 20 mM Tris-HCl, PH 8.0, 30 mM Na_4_P_2_O_7_, 40 mM NaCl, 5 mM EDTA, 1% NP-40, 2 mM Na_3_VO_4_, and 1 mM PMSF with a protease inhibitor cocktail (Sigma-Aldrich). The lysates (10–20 *μ*g total protein) were resolved by SDS-PAGE and analyzed by Western blotting. The blots were incubated for 60 min with a 1 : 400 dilution of primary rabbit anti-SOCS3 (Cell Signalling, Technology) or anti-*β*-actin antibody. The secondary antibody, HRP-conjugated donkey anti-rabbit IgG, was diluted 1 : 10000. Chemiluminescent protein bands were detected by an ECL detection system (Amersham Biosciences) according to the manufacturer's protocol.

### 2.5. Immunohistochemistry

The lungs were instilled with neutral buffered Formalin in PBS, pH 7.2, the trachea was tied off, and the lungs were immersed in Formalin overnight. After fixation, the lung tissues were embedded in paraffin and cut into 6-*μ*m-thick sections. Tissue sections (6 *μ*m) were deparaffinized with xylene and dehydrated with a series of graded alcohol solutions to automation buffer (AB) consisting of 5% NaCl and 2% HCl (Biomeda, Foster City, CA). Endogenous peroxidase was blocked in methanol and 3% (vol/vol) H_2_O_2_ for 15 min. After the sections were washed twice, blocking was performed with BSA 4% and normal goat serum 6% in PBS % 20 min at 25°C. The slides were incubated with a primary rabbit anti-SOCS3 antibody (Cell Signalling, Tech) at 60 *μ*g/mL for 30 min at room temperature. For detection of SOCS3, a rabbit Elite kit (Vector Laboratories, Burlingame, CA) was used as follows: sections were washed twice with AB and then incubated for 30 min with a 1 : 400 dilution of biotinylated secondary goat antirabbit IgG. Slides were washed again and incubated with the Elite avidin-biotin complex (Vector Laboratories) for 30 min. For staining, slides were washed 5 times with AB, and then the antibody complex was visualized using a diaminobenzidine tablet (10 mg; Sigma Chemical, St. Louis, MO) dissolved in 20 mL ofAB containing 12 *μ*L of 30% H_2_O_2_ for 6 min in the dark. All slides were then rinsed in running tap water, counterstained with hematoxylin (Harelco, Gibbstown, NJ), dehydrated through a series of graded alcohols to xylene, and covered with a coverslip with Permount (Fisher Scientific, Fair Lawn, NJ).

### 2.6. Statistical Analysis

The data from real-time RT-PCR were expressed as geometric mean and standard deviation (SD) values for a group size of 10–20 from three different experiments. Results were compared and evaluated using the Kruskal-Wallis test and Dunn's posttest multiple comparison test. Statistical significance was recognized at *P* ≤ .05. Statistical analyses were performed using GraphPad InStat3 (GraphPad Software Inc., San Diego, CA, USA).

## 3. Results

### 3.1. Differential Gene Expression

We used microarrays to profile expression of over 37,169 genes in lung tissues from six mice with asthma (OVA group), 8 mice treated with plasmid encoding Gal-3 gene (pEGFP-Gal-3 group), and five from the negative control group (SS group). Differential gene expression in each group was compared with that observed in other groups. OVA mice exhibited higher mRNA expression of TGF-*β*, SOCS-1, SOCS-3, IL17A, and IL17F than that seen in the pEGFP-GAL-3 mice. When comparing the pEGFP-Gal-3 and the SS groups, only 2 genes were uprepresented while for the OVA and the SS groups, 977 were uprepresented and 788 downrepresented (adjusted *P*-value < .05).

### 3.2. Gene Ontology

**Figure 1:**
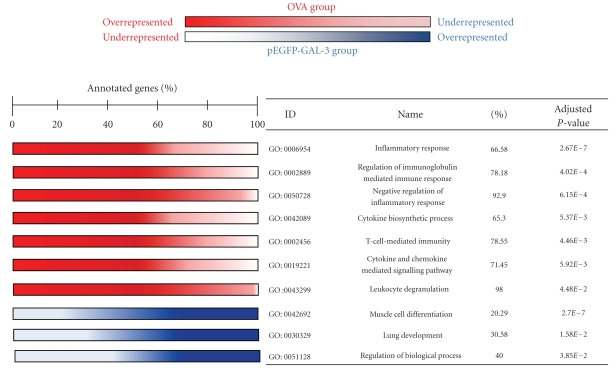
Biological pathway up- and downregulated in OVA versus pEGFP-GAL. Red indicates the biological processes overrepresented in the OVA group (percentage >50), and blue indicates that these pathways are overrepresented in the pEGFP-Gal-3 group (percentage **<**50).

The FasScan test for gene set enrichment analysis was applied to analyze the transcriptional profiles of mice treated with pEGFP-Gal-3 versus the untreated OVA group. We observed a significant upregulation of several biological processes and a downregulation of another (adjusted *P*-value <.05, Figure 1) at level 4 of gene ontology. 

Additional analysis of gene biological function revealed gene enrichment in several pathways. Interestingly, some of these pathways presented an overexpression of core genes in OVA mice and underexpression in Gal-3-treated mice (SOCS, TGF-*β*, and IL-10). Table 1 shows the differentially expressed genes within these functional categories.

### 3.3. Downregulation of SOCS1 and SOCS3 Gene Expression by Gal-3 Treatment

Considering the results obtained by microarray, we set out to discover whether Gal-3 treatment affected SOCS expression. SOCS1, SOCS3, and SOCS5 gene expression levels were analyzed by real-time quantitative PCR in lung tissue from mice groups. 

**Figure 2:**
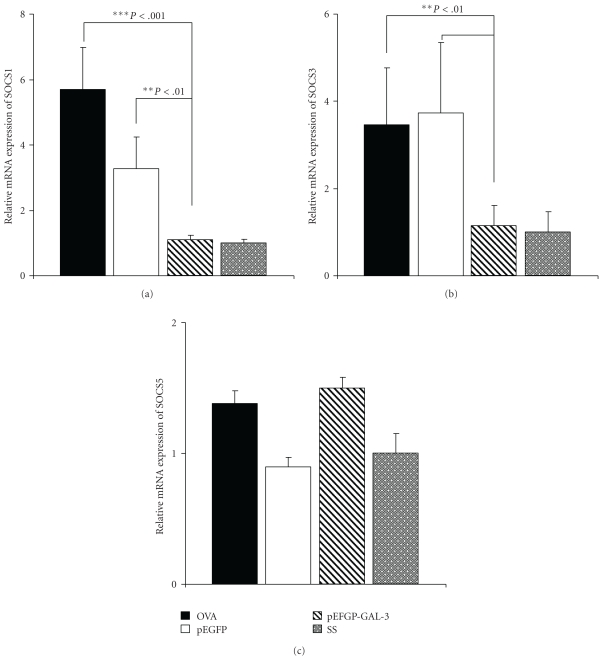
Semiquantitative expression of SOCS1, SOCS3, and SOCS5 genes in lungs after twelve weeks of OVA exposure. Relative mRNA levels of SOCS1 (a), SOCS3 (b), and SOCS5 (c) gene expression in lungs of different groups of mice were determined by real-time quantitative PCR. Values were normalized with rRNA gene used as endogen. Black bars represent the OVA group (*n* = 15), white bars the pEGFP group (*n* = 15), shaded bars the pEGFP-Gal-3 group (*n* = 20), and gray bars the SS group (*n* = 10). The results show relative gene expressions, as determined by the ΔΔC_T_  method, after twelve weeks of antigen exposure. Significant differences in SOCS3 and SOCS1 (**P < .01  and ***P < .001) expression levels were obtained for the pEGFP-Gal-3 versus the OVA and pEGFP groups.

When mice were treated for 12 weeks with Ag and pEGFP-Gal-3, the relative level of SOCS1 and SOCS3 expression was significantly lower than it was in OVA-exposed mice without gene treatment: 1.103 versus 5.69 *P* < .001 and 1.14 versus 3.47 *P* < .01, respectively, representing nearly 80.6% and 67.15% inhibition (Figures 2(a) and 2(b)). However, treatment with empty plasmid did not significantly modify SOCS expression in comparison with the OVA group. Moreover, statistically significant differences were observed upon comparing groups treated with empty plasmid and plasmid-encoding Gal-3 in SOCS1 and SOCS3 expression, respectively (*P* < .01).

There was no significant change in the levels of SOCS5 gene expression (Figure 2(c)).

### 3.4. Forced Gal-3 Expression by Gene Therapy Inhibits SOCS3 Protein Expression

**Figure 3:**
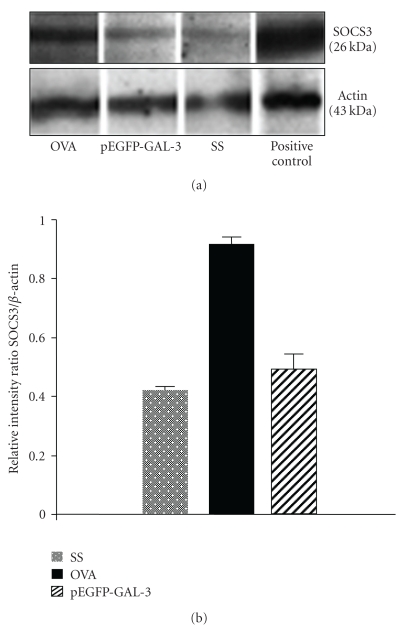
Western blot of SOCS3. (a) Protein extract from lungs was separated by 12% SDS-PAGE and blotted onto nitrocellulose membranes. Detection of SOCS3 was performed with goat antimouse IgG. Actin was used as the internal control. (b) SOCS3 bands were quantified by densitometry and corrected by actin expression. Densitometric analysis reveals a strong expression of SOCS3 protein in the OVA group, compared with the pEGFP-Gal-3 and SS groups. Data are expressed as the geometric mean ± SD, *n* = 4, *P < .05.

To ascertain whether the same effect was observable in protein expression, we performed a Western blot analysis in lung tissue using an anti-SOCS3 antibody. As depicted in Figure 3(a), higher SOCS3 protein expression was detected in the lungs of the OVA group of mice. The pEGFP-Gal-3 and SS groups showed low constitutive expression of SOCS3.  Figure 3(b) shows SOCS3 protein expression quantified by densitometry and normalized by *β*-actin. The results indicate that SOCS3 was more strongly expressed in the lungs of asthmatic mice than in the plasmid encoding Gal-3 gene and negative control mice.

### 3.5. Accumulation of SOCS3-Expressing Cells in the Lungs of Asthmatic Mice Is Inhibited by Gal-3

**Figure 4:**
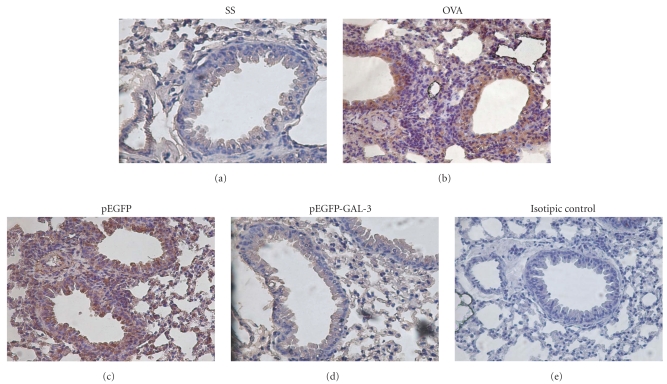
Immunohistochemical expression of SOCS-3 in lung tissue from A/J mice. (a) Saline control group (untreated). (b) Positive control group (OVA group) with chronic allergic airway inflammation. (c) pEGFP group, OVA immunized animals treated with empty plasmid. (d) pEGFP-Gal-3 group, OVA immunized animals treated with plasmid encoding Gal-3. (e) Control using normal goat IgG instead of goat anti-SOCS3 antibody. The picture is a representative example of 5 mice, all displaying similar results.

Accordingly, immunohistochemical analysis was then used to examine SOCS3 protein expression at the inflammatory site. In the OVA- and pEGFP-treated mice, SOCS3-expressing cells were present in high numbers at the inflammatory site (Figures 4(b) and 4(c)) and in airway epithelial cells. Whereas the Gal-3-treated mice displayed no SOCS3-expressing cells (Figure 4(d)), the appearance of the lungs was similar to that of the control mice (Figure 4(a)).

### 3.6. SOCS1 and SOCS3 Affect Production of Both IL-10 and TGF-*β*_1_ after Gene Inhibition with Gal-3

**Figure 5:**
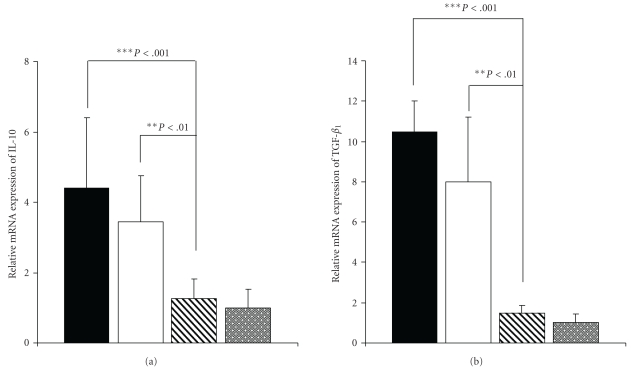
Effect of Plasmid with Gal-3 on Quantitative Expression of Cytokine Genes in lungs after Twelve Weeks of OVA Exposure. Relative mRNA levels of IL-10 (a) and TGF-*β*_1_(b) gene expression in lungs from different groups of mice were determined by real-time quantitative PCR. Values were normalized with the rRNA gene used as an endogen. Black bars represent the OVA group (geometric mean ± SD, *n* = 15), white bars the pEGFP group (geometric mean ± SD, *n* = 15), shaded bars the pEGFP-Gal-3 group (geometric mean ± SD, *n* = 20), and gray bars the SS group (geometric mean ± SD, *n* = 10). The results show relative gene expressions, as determined by the ΔΔC_T_  method, after twelve weeks of antigen exposure. Significant differences in IL-10 and TGF-*β*_1_ (***P < .001) expression levels were obtained for the pEGFP-Gal-3 versus the OVA and pEGFP groups. In the case of empty plasmid, no significant differences were found with respect to the OVA group.

Activated T cells migrate to the lungs and release inflammatory and regulatory cytokines to orchestrate the allergic response. Given the differential SOCS3 expression in groups of mice (Gal-3-treated versus OVA), we predicted that regulatory cytokines would be affected by the relatively low levels of SOCS1 and SOCS3 expression in Gal-3-treated mice. We, therefore, assessed the effect of Gal-3 gene therapy on regulatory cytokine levels (IL-10 and TGF-*β*_1_) in lungs after 12 weeks of chronic OVA-challenge. When the mice were treated for twelve weeks with antigen and pEGFP-Gal-3, the relative level of IL-10 and TGF-*β*_1_ expression was significantly lower than it was among OVA-exposed mice without gene treatment: 1.2 versus 4.4 and 1.4 versus 10.5 *P* < .001, representing nearly 72.72% and 86.60% inhibition, respectively (Figure 5). However, treatment with empty plasmid did not significantly modify IL-10 and TGF-*β*_1_ expression compared to the OVA group.

## 4. Discussion

The present study used microarray technology to define the patterns of expression associated with Gal-3 gene therapy in lungs of asthmatic mice. In particular, we compared gene expression in lungs from OVA and pEGFP-Gal-3 groups. We identified a “Gal-3 treated signature” gene-expression profile that was induced in the OVA mouse lung by exposure to Gal-3. This signature profile included several genes related to lung development, inflammatory response, and so forth. One of the most important findings of this study is the failure to identify a sole induced gene despite the fact that several pathways were indeed induced or underregulated. This is not surprising because asthma is a complex disease with many factors.

Traditional strategies for gene expression analysis have focused on identifying individual genes that exhibit differences between two states of interest. Although useful, they fail to detect complex biological processes that are distributed across a network of genes and are subtle at the level of individual genes. Also, high variability between mice and limited sample sizes make it difficult to distinguish gene differences. Thus, an analytical strategy, Gene Set Enrichment Analysis, has been designed to detect coordinate changes in the expression of groups of functionally related genes [25]. 

The analysis of gene biological function revealed gene enrichment in several pathways when comparing the OVA and pEGFP-Gal-3 groups. Interestingly, inflammatory response, leukocyte activation, and cytokine production processes presented a significant overexpression in mice from the OVA group compared to mice treated with Gal-3 gene therapy. These data were consistent with our previous results [16]. One of the pathways that underwent significant enrichment in OVA mice in comparison to the pEGFP-GAL-3 mice group were the processes implicated in negative regulation of inflammatory response. Prominent among this class of intracellular regulators are members of the SOCS family of proteins [3, 26]. Significant interest in the SOCS family stems from the belief that SOCS proteins may integrate multiple cytokine signals and mediate cross-communication between antagonistic cytokines produced by different cells through their inhibitory effects on cytokine receptors and signaling molecules. 

SOCS proteins exercise negative regulation for many aspects of cytokine signaling. Hence, some members of the SOCS protein family (SOCS1, 3 and 5) participate in the regulation of Th1/Th2 balance [27, 28]. 

We confirmed microarray data using real-time quantitative PCR, demonstrating that mice treated with Ag and pEGFP-Gal-3 showed significantly lower levels of SOCS1 and SOCS3 expression than OVA-exposed mice not receiving gene treatment. These data are in concordance with other studies described in the literature in which no differences were found in SOCS5 gene-expression levels between healthy patients and those with asthma or atopic dermatitis. Nevertheless, SOCS3 expression levels are high in T cells from patients with this allergic disease [29].

In the group of mice receiving plasmid with Gal-3 gene by intranasal administration, we obtained values similar in SOCS3 and SOCS1 gene expression to those of the healthy group and lower than those of the other two groups of asthmatic mice that had not received pEGFP-Gal-3. This suggests that gal-3 may be exercising some direct or indirect effect on the transcription of the genes of SOCS3 and SOCS1. 

The impact of gal-3 therapy on SOCS3 was further confirmed by Western blot and by immunohistochemical analysis. Accumulation of SOCS3-expressing cells at the inflammatory site coincides with accumulation of inflammatory cells. In the mice treated with Gal-3, however, there was a correlation between non-SOCS3-expressing cells and absence of inflammatory cells, probably due to the fact that SOCS3 is predominantly expressed in Th2 cells. These results suggest that migration of SOCS3-expressing cells may be implicated in the development and exacerbation of lung inflammation, therefore suggesting that downregulation of SOCS3 expression by Gal-3 inhibits development of lung inflammation. In brief, the protein expression results were similar to those yielded by gene expression analysis.

Activated T cells migrate to the lungs and release inflammatory and regulatory cytokines to orchestrate the allergic response. We, therefore, assessed the effect of Gal-3 gene therapy on lung regulatory cytokine levels (IL-10 and TGF-*β*_1_) after 12 weeks of chronic OVA-challenge. Our results indicated that IL-10 and TGF-*β*_1_ may be intermediary cytokines involved in SOCS3 expression. Also appearing in Table 1 are *IL17A, IL17B, IL17F, TGFB1, FOXP3, IL10, SOCS3, SOCS1, SOCS5,* and* STAT3* as genes that are up-regulated in Gene Ontology pathways in OVA versus pEGFP-GAL 3 mice, all of which are implicated in regulatory immune response. In a finding similar to ours, McGee et al. observed that the therapeutic effect of FMS-like tyrosine kinase 3 (Flt3) in reversing the hallmarks of allergic asthma in a mouse model is mediated by decreasing TGF-*β*, which in turn decreases Th17 cells and the expression of SOCS1 and SOCS3 in the lung of asthmatic mice [30]. Also, there is increasing evidence showing that IL-10 induces SOCS3 gene expression through the activation of STAT3, which activates the SOCS3 promoter [31]. 

This study demonstrates that SOCS proteins are implicated in the mechanism whereby Th2 allergic asthma is inhibited by Gal-3. The role of Gal-3 in TH1/Th2 immune and inflammatory responses appears to vary according to the experimental models used (administration of Gal-3 versus Gal-3 null animals) [15, 16, 32]. We provide evidence of a correlation between treatment with Gal-3 and inhibition of SOCS1 and SOCS3 expression in lungs. These results suggest that negative regulation of SOCS1 and 3 by Gal-3 treatment could be a good therapeutic approach in allergic diseases. 

Accordingly, one of the new models of therapy for allergic diseases could entail targeting SOCS3 by using a Gal-3 gene therapy approach via inhibition of Th2 response. Such an immunomodulatory approach may have the beneficial effect of controlling the appropriate balance of the immune system.

## Figures and Tables

**Table 1:** Genes included in upregulated gene ontology pathways in OVA versus pEGFP-GAL 3 comparison.

Biological process (GO database)	Genes
Inflammatory response	*IDO1, **IL17A**, **IL17B**, **IL17F**, IL1A, IL25, **TGFB1**, IL6, IL4, **FOXP3**, JAK2, STAT5A, TLR4*
Regulation of immunoglobulin mediated immune response	*BCL6, FOXP3, FCG2B, IGH7, TNF, **TGFB1**, IL4, STAT6, IGH1A, IGH3, IGH7, C3, IL27RA*
Negative regulation of inflammatory response	***IL10**, **FOXP3**, **TGFB**, **SOCS3**, IL2, TNF1B, AD, FOXF1A, IL2RA, ZFP36*
Cytokine biosynthetic process	*ADAM3, GATA3, IGF2BP1, IL12B, MAP2K3, IL6, **FOXP3**, **IL-10**, NFKB1, BCL3, INFG, IL1B, TNF, WNT5A, IL21, IL1A, STAT5, **IL17F***
T-cell-mediated immunity	*IL4RA, BCL6, **TGFB**, IL20RB, IL2RA, IDO, **FOXP3**, CD27, CD74, LAG3, CASP3 *
Cytokine and chemokine mediated signalling pathway	*ILR1, IL23R, IL2RB, IL3, IL5, JAK1, JAK3, **SOCS1**, **SOCS3**, **SOCS5**, STAT1, **STAT3**, STAT5A, STAT5B, IL6, IL6RA, TNF *
Leukocyte degranulation	*P4K2A, RAB27A, IGH7, HMOX1, FOXF1A, FCER1A, FCER1G, PRAM1*

GO: Gene Ontology.
